# Post-Transcriptional Regulation of *BCL2* mRNA by the RNA-Binding Protein ZFP36L1 in Malignant B Cells

**DOI:** 10.1371/journal.pone.0102625

**Published:** 2014-07-11

**Authors:** Anna Zekavati, Asghar Nasir, Amor Alcaraz, Maceler Aldrovandi, Phil Marsh, John D. Norton, John J. Murphy

**Affiliations:** 1 Division of Immunology, Infection and Inflammatory Disease, King's College London, London, United Kingdom; 2 Department of Biomedical Sciences, University of Westminster, London, United Kingdom; 3 Division of Endocrinology, King's College London, London, United Kingdom; 4 School of Biological Sciences, University of Essex, Colchester, Essex, United Kingdom; University of Hawaii Cancer Center, United States of America

## Abstract

The human ZFP36 zinc finger protein family consists of ZFP36, ZFP36L1, and ZFP36L2. These proteins regulate various cellular processes, including cell apoptosis, by binding to adenine uridine rich elements in the 3′ untranslated regions of sets of target mRNAs to promote their degradation. The pro-apoptotic and other functions of ZFP36 family members have been implicated in the pathogenesis of lymphoid malignancies. To identify candidate mRNAs that are targeted in the pro-apoptotic response by ZFP36L1, we reverse-engineered a gene regulatory network for all three ZFP36 family members using the ‘maximum information coefficient’ (MIC) for target gene inference on a large microarray gene expression dataset representing cells of diverse histological origin. Of the three inferred ZFP36L1 mRNA targets that were identified, we focussed on experimental validation of mRNA for the pro-survival protein, BCL2, as a target for ZFP36L1. RNA electrophoretic mobility shift assay experiments revealed that ZFP36L1 interacted with the *BCL2* adenine uridine rich element. In murine BCL1 leukemia cells stably transduced with a ZFP36L1 ShRNA lentiviral construct, *BCL2* mRNA degradation was significantly delayed compared to control lentiviral expressing cells and ZFP36L1 knockdown in different cell types (BCL1, ACHN, Ramos), resulted in increased levels of *BCL2* mRNA levels compared to control cells. 3′ untranslated region luciferase reporter assays in HEK293T cells showed that wild type but not zinc finger mutant ZFP36L1 protein was able to downregulate a *BCL2* construct containing the *BCL2* adenine uridine rich element and removal of the adenine uridine rich core from the *BCL2* 3′ untranslated region in the reporter construct significantly reduced the ability of ZFP36L1 to mediate this effect. Taken together, our data are consistent with ZFP36L1 interacting with and mediating degradation of *BCL2* mRNA as an important target through which ZFP36L1 mediates its pro-apoptotic effects in malignant B-cells.

## Introduction

The human ZFP36 protein family consists of three widely-expressed members, namely, ZFP36 (TIS11, TTP, Nup475, GOS24), ZFP36L1 (Tis11b, Berg36, ERF-1, BRF-1), and ZFP36L2 (Tis11d, ERF-2, BRF-2) [1]–[3]. A fourth family member described in rodents, Zfp36l3, displays placental-specific expression, but is not detectably expressed in any human tissues [4]. ZFP36-family proteins have also been identified and characterised in some other species such as Xenopus, *Drosophila* and yeast [5]–[7]. These proteins contain two tandemly repeated zinc finger motifs and function to regulate gene expression at the post-transcriptional level by binding to adenine uridine (AU) rich elements (AREs) in the 3′ untranslated region (3′UTR) of sets of mRNAs and mediating ARE-dependent mRNA decay [1]–[3]. In mammals, ZFP36 family members have been shown to function in regulating development, cell differentiation, tumourigenesis, the inflammatory response and apoptosis by targeting an extensive overlapping repertoire of mRNAs. These have been best characterised in the inflammatory/immune response in which all three ZFP36 family members elicit rapid downregulation of key cytokines via destabilisation of their mRNAs (reviewed in [1]–[3]). Members of the ZFP36 family also target mRNAs encoding key transcription factors, such as STAT5b in the regulation of erythropoiesis [8] and PRDM1/Blimp1 in terminal plasmacytoid differentiation of B cells [9].

We originally reported on the pro-apoptotic function of ZFP36L1 in Ramos Burkitt B lymphoma cells [10] and more recently in Rituximab-induced apoptosis of B-chronic lymphocytic leukaemia cells (BCLL) [11] from which the human *ZFP36L1* gene was originally isolated as an early response gene cDNA [12]. Overexpression of ZFP36 family members has been shown by other laboratories to induce apoptosis in a variety of other mammalian cell lines including HeLa, U20S, SAOS2, and 3T3 [13], [14]. Induction of apoptosis by all three ZFP36 family members is completely abrogated in the presence of Bcl-2 or CrmA [13]. ZFP36 synergistically induces apoptosis with TNF-α in 3T3 cells and the zinc fingers and the N-terminal domain of ZFP36 are absolutely required for this effect [14]. Mutant ZFP36 (TIS11) lacking the zinc finger motifs fails to induce apoptosis and is localised to the nucleus, whereas the wild type ZFP36/TIS11 is localised in the cytoplasm [14]. Induction of apoptosis by ZFP36 family proteins therefore appears to require intactness of the zinc finger motifs and presumably mRNA binding. However, the identities of mRNAs that are targeted by ZFP36 family members in mediating their pro-apoptotic effects are currently unknown.

To identify candidate mRNAs that are targeted in the pro-apoptotic response by ZFP36L1, we reverse-engineered a gene regulatory network for ZFP36 family members using the ‘maximum information coefficient’ (MIC) for target gene inference [15] Of the final set of three inferred anti-apoptosis ZFP36L1 targets identified by this analysis, we focussed on experimental validation of mRNA for the well-characterised BCL2 protein and here present evidence that *BCL2* mRNA is a target for ZFP36L1 protein. Targeting *BCL2* mRNA, and potentially other anti-apoptotic mRNAs, may explain at least in part the reported pro-apoptotic effects of ZFP36L1 protein.

## Materials and Methods

### Data mining of microarray expression gene expression data

To identify mRNAs that are regulated by ZFP36 family members at a global level, a gene expression microarray dataset comprised of 206 cell line samples representing cell types of diverse histological origin was downloaded from NCBI Gene Expression Omnibus database (Affymetrix; GSE10843, http://www.ncbi.nlm.nih.gov/geo). The data was fRMA-normalised [16] and barcode-filtered [17] to generate probeset values representing 10016 unique protein-coding genes. An unsupervised reverse engineering of gene regulatory networks approach was employed to determine pair-wise statistical dependences between expression of each ZFP36 family member and all other genes in the dataset. Algorithms used were Maximum Information Coefficient (MIC), Mutual Information (MI) and Linear Regression (LR). MIC and LR values were computed using the maximal information-based nonparametric exploration (MINE) statistics package in Bioconductor, R [15], using a threshold cut-off MIC score corresponding to a false discovery rate (FDR) q value (calculated using 3×10^7^ permutations) of 0.01. MI values were computed using ‘ARACNE’ (Algorithm for the Reconstruction of Accurate Cellular Networks) [18], v2 with a data processing inequality tolerance (DPI) value of 1.0 as implemented in the GenePattern (v3.6.0) suite of software tools [19]. Network inference performance was evaluated/validated by gene set enrichment analysis (GSEA) [20] on inferred ZFP36 target genes, ranked by inference metric, using two independent gene sets representing experimentally-identified ZFP36 target genes in fibroblasts from *ZFP36/Tis11*-knock-out mice. The first of these [21] comprised a 237 gene set for transcripts that are significantly (FDR<0.01) up-regulated in *ZFP36/Tis11*-knock-out *versus* wild type fibroblasts, while the second comprised a 152 gene set representing transcripts that display a decreased rate of decay (stabilisation) in *ZFP36/Tis11*-knock-out fibroblasts after serum stimulation and actinomycin D treatment in time-course microarray experiments [22]. Genes displaying a significant increase in stabilisation in *ZFP/Tis11*-knock-out cells were identified using the maSigPro algorithm [23] in Bioconductor R on the microarray dataset, GSE5324 [22] using a FDR cut-off value of 0.01.

MIC-inferred target genes for all three ZFP36 family members were filtered using the ‘ARED’ database [24] for the presence of two or more copies of the consensus 13 bp AU-rich element motif: WWWU(AUUUA)UUUW in the 3′-untranslated region (UTR) that is present in most characterised ZFP36/Tis11 target mRNAs [25], [26]. Target genes were further filtered by the sign of the LR coefficient to identify those whose expression is inversely correlated with each of the ZFP36 family members (candidates for targeted degradation). Gene Ontology (GO) enrichment analysis was performed on the final combined list of ZFP36 family targets using the GSEA on-line database (http://www.broadinstitute.org/gsea/index.jsp) [20]. Network graphs were constructed and visualised in Cytoscape v2.8 [27]. Statistical analysis of gene overlap by hypergeometric distribution was performed using the ‘phyper’ algorithm in Bioconductor R.

### Cell Culture

Murine B cell leukemia BCL1 cells were obtained from the European collection of cell cultures (ref. No. 90061904) and grown in RPMI 1640 medium supplemented with 10% FBS, 2 mM L-Glutamine, 50 U/mL penicillin streptomycin, 1% sodium pyruvate, 1% non-essential amino acids and 0.05 mM 2-mercaptoethanol. Ramos human Burkitt lymphoma B cells (European collection of cell cultures ref. No. 85030802) and ACHN human renal carcinoma cells (NCI-Frederick DCTD Tumor/Cell Line Repository, 0503808) were grown in RPMl 1640 medium (Invitrogen) supplemented with 10% FBS, 50 U/mL penicillin streptomycin, and 2 mM L-Glutamine. All cells were cultured at 37°C, in a humidified CO_2_ incubator. For some experiments, Ramos cells were serum starved in FBS-free medium for 16 hours and stimulated with 30 nM of Phorbol 12-Myristate 13-Acetate (PMA) for 3 hours.

### Modulation of ZFP36L1 levels using a ShRNA expressing lentivirus in different cell types

A ZFP36L1-ShRNA lentivirus was constructed by cloning a ZFP36L1 ShRNA into the pSicoR lentiviral vector [28] as outlined previously [9]. Stable mammalian cell lines containing the ZFP36L1-ShRNA, empty lentivirus or a scrambled version of the ZFP36L1-ShRNA were prepared following transduction of cells with virus and cell sorting GFP positive cells on a flow cytometer (BCL1 and Ramos cells). Transduction efficiency was very high for ACHN cells (>95%) and no cell sorting was required. Efficiency of ZFP36L1 knockdown was assessed by qRT-PCR and Western Blot analysis. For details on oligonucleotides designed for ZFP36L1 ShRNA and scramble sequence please see [9].

### Bacterial Expression of ZFP36L1 and protein purification using Ni2+-Affinity Chromatography

Human ZFP36L1 protein was expressed in *E.coli* and purified using a protocol similar to one previously published [29]. A cDNA encoding the open reading frame of human *ZFP36L1* was cloned into pET15b plasmid. Overnight cultures of *E.coli* BL21 (DE3) (Agilent, Stockport, UK) transformed with recombinant plasmids were harvested, the cell pellet went through one cycle of freezing at −70°C and thawing at 37°C and was resuspended in 20 ml of lysis buffer (5 mM Imidazole, 0.5 M NaCl, 20 mM NAPO4, 1 mg/ml Lysosyme, 1 mM PMSF, and 5 U/ml DNase; pH 7.4). The cells were disrupted by a brief sonication and their pellets containing inclusion bodies were resuspended overnight at 4°C in 100 ml of denaturing buffer (8 M Urea, 0.5 M NaCl, and 20 mM NAPO4; pH 8.0). Solubilized denatured proteins were recovered in the supernatant following centrifugation at 18 000 rpm for 15 min at 4°C. Using a Pharmacia LKB Pump P-1 (GE Healthcare, Bio-Sciences AB, Uppsala, Sweden), a HiTrap Chelating column (GE Healthcare) was washed with 300 ml DH2O, charged with 30 ml of 0.1 M NiCl charging buffer, and again washed with 100 ml of deionised water. The column was equilibrated in 100 ml of denaturing buffer (8 M Urea, 0.5 M NaCl, and 20 mM NAPO4; pH 8.0) and the urea-solubilized proteins were loaded onto the nickel-conjugated column to allow binding of the His-tagged protein to the nickel column. 100 ml of buffer A (20 mM Imidazole, 8 M Urea, 0.5 M NaCl, and 20 mM NAPO4; pH 8.0) was used to wash the column. Bound proteins were refolded on the column by slowly decreasing the urea concentration of denaturing buffer from 8 M to 0 M urea (8 M, 6 M, 4.5 M, 3 M, 2 M, 1 M, 0.5 M, 0.2 M, 0.1 M, 0 M). A gradient volume of 20 ml and a flow rate of 1 ml/min were used for refolding step. The refolded recombinant protein was eluted with EDTA buffer (500 mM NaCl, 20 mM NAPO4, and 50 mM EDTA, and 1× Roche Protease Inhibitor Cocktail; pH 7.4) after the gradient has come to its endpoint. The eluted protein was dialysed at 4°C against 3 L of PBS (pH 7.4). Protein concentration was measured by a Bicinchoninic protein assay (Sigma, Poole, UK).

### Western blotting

Cells were washed with ice-cold PBS and lysed on ice for 30 min in 100 µl of Nonidet P-40 buffer (1% NP - 40, 20 mM Tris HCl (pH 7.4), 150 mM NaCl, 2 mM MgCl2, and 1 mM EDTA) containing Complete Protease Inhibitor Cocktail (Roche) and 1 mM of phenylmethylsulfonyl fluoride. Cytoplasmic protein extract was separated from nuclei by centrifugation at 13 000 RPM at 4°C for 10 min. Protein extracts were separated by SDSPAGE Electrophoresis and electrotransferred onto nitrocellulose membranes which were blocked overnight at 4°C in blocking buffer containing 5% milk powder and TTBS buffer (23 mM Tris base, 0.5 M NaCl, and 0.05% Tween-20). Nitrocellulose membranes were incubated with appropriate dilution of the following primary antibodies for 1 hour; purified rabbit polyclonal anti-ZFP36L1/L2 (cat. no. 2119, Cell Signaling, Danvers, MA, USA), purified rabbit anti-BCL2 antibody (cat. No. #2876, Cell Signalling Technology, MA, USA)) or purified rabbit anti-human HSP90 (cat. no. SC-7947, Santa Cruz BiotechnologySanta Cruz, CA, USA) as a loading control. Following this incubation, membranes were washed and incubated with horseradish peroxidise-conjugated swine anti-rabbit immunoglobulin for 1 hour. Chemiluminescent signals on membranes were detected using SuperSignal West Femto Substrate (Pierce, Rockford, IL, USA) and exposed to XRayfilms (Kodak Biomax MS, Sigma). The films were developed in a Xograph Compactx4 film processor (Xograph Imaging Systems Ltd. Tetbury, UK).

### RNA Electrophoretic Mobility Shift Assay (REMSA)

^32^P-labeled and unlabeled mRNA probes were made *in vitro* using Riboprobe *In Vitro* Transcription Systems (Promega, Southampton, UK) in the presence or absence of 40 μCi of [α-^32^P]rUTP (GE Healthcare) respectively. The *BCL2* ARE probe was generated using the plasmidPCRII/*bcl-2* ARE [30]. The resulting probe was separated from unincorporated nucleotides using ProbeQuant G-50 Micro Columns (GE Healthcare). *E.coli*-expressed purified ZFP36L1 protein (10–100 ng) or cell protein cell lysates (30 µg) were incubated with 50 000 to 100 000 cpm of α-^32^P-labeled RNA probes, corresponding to approximately 30–100 fmol RNA. The proteins were incubated with RNA probes for 20 min on ice in RNA-binding buffer containing 20 mM HEPES (pH 7.6), 3 mM MgCl2, 40 mM KCl, 2 mM DTT, and 5% Glycerol in a total volume of 20 µl. RNase T1 and heparan sulfate (Sigma) were added to final concentrations of 50 U/ml and 5 mg/ml, respectively, and incubated on ice for another 20 min. RNA-protein complexes were resolved by electrophoresis (150 V for 3 hours at 4°C) on a 4% nondenaturing polyacrylamide gel using a Hoefer SE600 electrophoresis unit (Hoefer Scientific, Holliston, MA, USA). The gel was dried on a gel dryer (Hoefer SE1160 Gel Dryer), exposed to X-ray film (Kodak BioMax MS) and developed.

### Quantitative RT-PCR analysis

Total RNA was extracted using an RNeasy Mini Kit (Qiagen, Crawley, UK). 1 µg of total RNA was reverse-transcribed for cDNA synthesis using a QuantiTect Reverse Transcription kit (Qiagen). Quantitative RT-PCR was performed using an ABI Prism 7000 Sequence Detection System (Applied Biosystems, Carlsbad, CA, USA), in a total volume of 25 µl containing cDNA, 2× QuantiTect SYBR Green Master mix (Qiagen), and 0.5–1 µM of each oligonucleotide primer. The primer pairs used for the following genes were: mouse *Bcl-2* QuantiTect primer set QT00156282 (Roche, Basel,

Switzerland), mouse *zfp36l1* forward 5′-TTCACGACACACCAGATCCT-3′ and reverse 5′-TGAGCATCTTGTTACCCTTGC-3′, mouse *IL-3* forward 5′-ATTCTACATGGTCCACCTTAACGA-3′ and reverse 5′-GGCTGAGGTGGTCTAGAGGTT-3′ mouse *β actin* forward 5′-CTAAGGCCAACCGTGAAAAG-3' and reverse 5'-ACCAGAGGCATACAGGGACA-3′, human *ZFP36L1* forward 5′-GATGACCACCACCCTCGT-3′ and reverse 5′-CTGGGAGCACTATAGTTGAGCA-3′, human *β actin* forward 5′-CCAACCGCGAGAAGATGA-3′ and reverse 5′-CCAGAGGCGTACAGGGATAG-3′, human *Bcl-2* forward 5′-AGTACCTGAACCGGCACCT-3′ and reverse 5′-GGCCGTACAGTTCCACAAA-3′. All PCR reactions were carried out in triplicate. The PCR program consisted of 1 initial cycle of 15 min at 95°C, 40 cycles of 15 sec at 95°C, 15 sec at 60°C, and 30 sec at 72°C. The relative changes in gene expression levels were calculated using the 2–ΔΔCT method [31].

### 3′UTR luciferase reporter assay

Human *BCL2* ARE sequences from plasmids PCRII/*bcl-2* ARE+ and PCRII/*bcl-2* mutARE [30] were cloned into the pmirGlo luciferase reporter vector (Promega) to generate *BCL2* ARE+ and *mutBCL2* ARE luciferase reporter vectors. *BCL2* ARE+ contains a more extensive region of *BCL2* 3′ UTR sequences surrounding the AU-rich core than *BCL2* ARE which was used in the REMSA analysis (see above) and *mutBCL2* ARE is similar to *BCL2* ARE+ but lacks the AU-rich core region present in *BCL2* ARE+ [30]. 24 h prior to transfection HEK 293T cells (2×10^5^ cells/ml) were seeded per well of a 12 well plate. Cells were then transfected with 100 ng of *BCL2* ARE probes in luciferase reporter vector with 200 ng of human ZFP36L1 expression vector (pcDNA6ZFP36L1) or ZFP36L1 with a zinc finger domain mutation (mutZFP36L1) or empty vector (pcDNA3). Cells were left for 24 h after transfection, then cell lysates were prepared, and luciferase and renilla signals measured using the Dual luciferase reporter assay system (Promega) on a Fluostar Optima (BMG, Labtech) plate reader.

### MTT assay to measure ionophore-induced Ramos cell viability

An MTT cell viability assay was carried out to measure calcium ionophore-induced cell death in Ramos cells under different conditions. ZFP36L1 siRNA oligonucleotide (Stealth RNAi, Invitrogen) and control siRNA (Stealth negative control RNAi, Invitrogen) were transfected into Ramos cells using Viromer blue transfection reagent (Lipocalyx, Halle, Germany) according to the manufacturer's instructions. 24 h later cells were treated with an optimum concentration of ionophore (A23187, 150 nM) [10] and then, after 20 h incubation, MTT (0.5 mg/ml) was added to cells. After 4 h incubation, MTT levels were measured on a spectrophotometer at 490 nm.

### *Ad hoc* Statistical Analysis

The Student T test was used to determine differences between samples. Values were expressed as mean ±SEM.

## Results

### Inference of ZFP36 apoptotic target mRNAs from gene expression data

**Figure 1:**
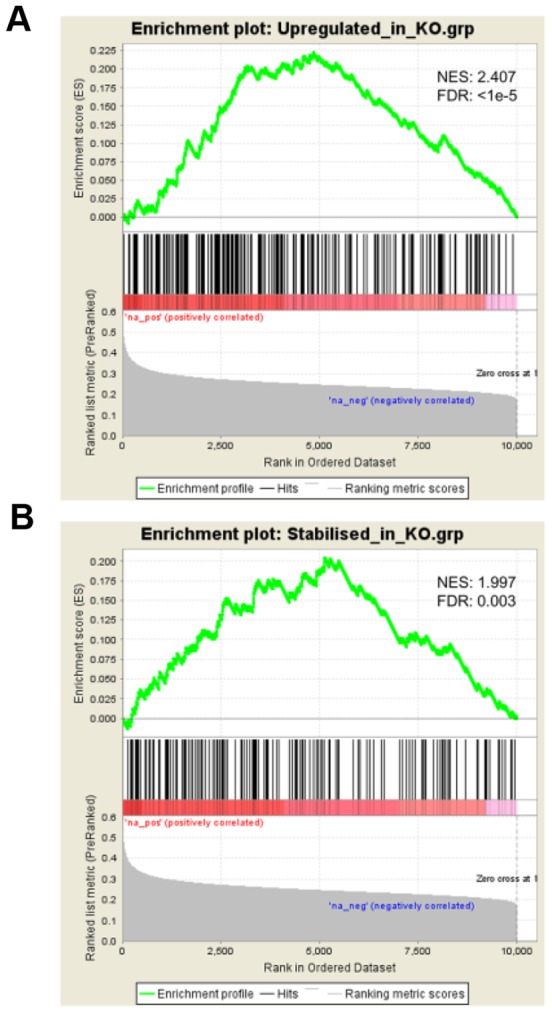
Validation of ZFP36 target genes inferred from MIC analysis of microarray gene expression data. Gene set enrichment analysis [20] profiles are shown for 10016 genes, rank-ordered by MIC score. Normalised Kolmogorov-Smirnov enrichment scores (NES) and false discovery rate q values (FDR) are shown. (A) using a gene set (n = 237) representing mRNAs that are significantly up-regulated in *Tis11/ZFP36*-knock-out mouse fibroblasts [21], (B) using a gene set (n = 152) representing mRNAs that display a significantly decreased rate of degradation in *Tis11/ZFP36*-deficient mouse fibroblasts [22].

To identify candidate mRNAs that are targeted in the pro-apoptotic response by ZFP36 proteins, we reverse-engineered a gene regulatory network for all three ZFP36 family members using three different algorithms (maximum information coefficient, MIC; mutual information, MI; linear regression, LR) to calculate pair-wise statistical dependency from a large microarray gene expression dataset. The performance of each algorithm in terms of accuracy/reliability was evaluated by gene set enrichment analysis (GSEA) between inferred ZFP36 targets and experimentally-identified ZFP36 targets represented by two independent gene sets obtained from Zfp36/Tis11 knock-out fibroblasts (see Materials and Methods). As shown in Table 1, the MIC algorithm [15] out-performed MI and LR calculations for target gene inference, with highly significant GSEA Kolmogorov-Smirnov statistical scores on each of the two independent experimental gene sets. Fig. 1 shows the GSEA profiles obtained with rank-ordered MIC-inferred ZFP36 targets. The MIC algorithm was therefore used for subsequent analysis on all three ZFP36 family members.

**Table 1:** Comparison between gene set enrichment validation results for ZFP36-inferred target genes obtained using different metrics of statistical-dependency.

METHOD	Up-regulated in KO^a^		Stabilised in KO^b^	
	**NES**	**FDR**	**NES**	**FDR**
MIC	2.407	<0.00001	1.997	0.003
MI	1.193	0.042	1.194	0.102
LR	1.098	0.086	0.960	0.645

Abbreviations: KO, *Tis11/ZFP36* knock-out mouse fibroblasts; NES, normalised enrichment score from Kolmogorov-Smirnov gene set enrichment analysis; FDR, false discovery rate *q* value from Kolmogorov-Smirnov gene set enrichment analysis; MIC, maximum information coefficient; MI, mutual information, LR, linear regression coefficient.

a Gene set representing 237 mRNAs displaying significant up-regulation in expression in fibroblasts from *Tis11/ZFP36*-deficient mouse fibroblasts [21].

b Gene set representing 152 mRNAs displaying significantly decreased rate of degradation in *Tis11/ZFP36*-deficient mouse fibroblasts [22].

**Figure 2:**
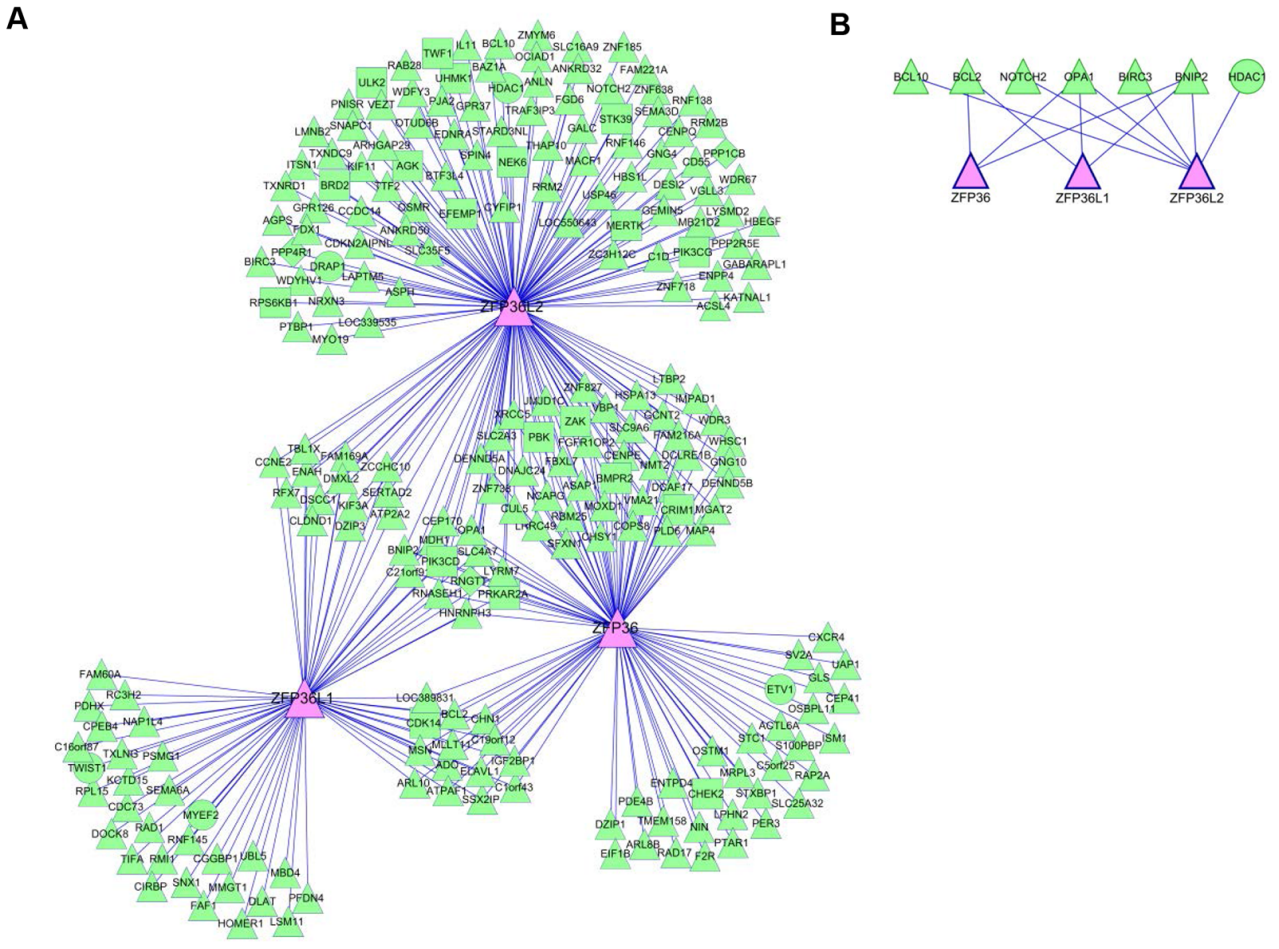
Reverse-engineering a gene regulatory network for ZFP36 family identifies *BCL2* mRNA as a direct target for ZFP36L1. (A) Network graph image of inferred target mRNAs for ZFP36 family members. Edges represent regulatory interactions inferred from significant pair-wise statistical dependences calculated from MIC scores (see Table S2). A force-directed Cytoscape layout is shown. Identification of *BCL2* mRNA as a direct target for ZFP36L1. (B) Candidate anti-apoptotic targets for ZFP36 family members identified from a reconstructed regulatory network. A hierarchical Cytoscape layout is shown.

After statistical thresholding and filtering for the subset of MIC-inferred targets that contain ARE elements in their 3′UTRs, the list of candidate target genes for ZFP36 family members (Table S1) was found to be over-represented in GO terms for several biological processes that included “apoptosis/programmed cell death” (Table S2). Fig. 2A shows a network graph view of the inferred target genes for each ZFP36 family member. While each ZFP36 family member was inferred to target its own set of unique mRNAs, there was a highly significant overlap of targets that were common in pair-wise comparisons of different ZFP36 family members (P = <1e-7, by hypergeometric distribution). The inferred target genes were finally filtered to identify those encoding proteins with GO terms that included “apoptosis/programmed cell death” (Table S2) that are known to negatively regulate programmed cell death. The resulting network graph depicting inferred pro-survival target mRNAs that are negatively regulated in the pro-apoptotic response of ZFP36 family members is shown in Fig. 2B. ZFP36 and ZFP36L1 share the same three targets (*BCL2, BNIP2, OPA1*) while ZFP36L2 shares *BNIP2* and *OPA1* with ZFP36/ZFP36L1 and also targets four additional mRNAs (*BCL10, NOTCH2, BIRC3, HDAC1*). For a full description of these inferred targets, see Table S1. Of the seven predicted targets, four (*BCL-2*, *BCL10*, *BIRC3*, *BNIP2*) function in well-defined apoptosis pathways while the other three predicted targets (*HDAC1*, *OPA1*, *NOTCH2*) have additional more general roles in controlling gene expression and/or developmental pathways.

### ZFP36L1 interacts with the *BCl2* ARE and mediates its degradation

**Figure 3:**
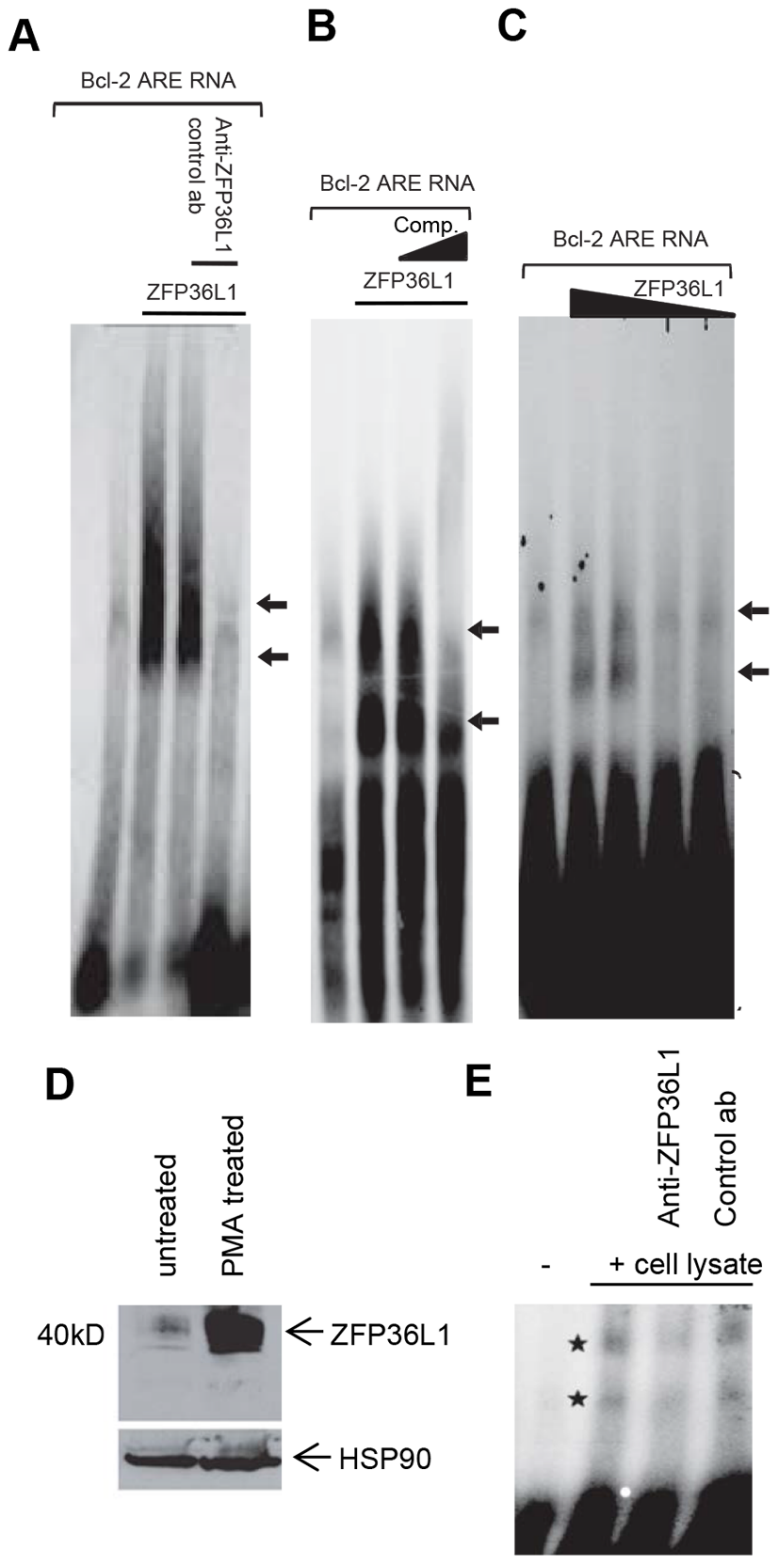
RNA electrophoretic mobility shift analyses of ZFP36L1 binding to the *BCL2* ARE. (A) REMSA analysis of recombinant ZFP36L1 binding to *BCL2* ARE RNA probes (see Methods). 50 ng of purified recombinant ZFP36L1 protein was incubated with ^32^P-labeled *BCL2* ARE. The specificity of RNA-protein complexes were examined by addition of 80 ng of anti-ZFP36L1 antibody (Cell Signaling Technology rabbit-anti-human ZFP36L1/L2 antibody) or a non-specific control (rabbit IgG) for 10 min prior to addition of radiolabeled probes as indicated (B) REMSA analysis of recombinant ZFP36L1 binding to *BCL2* ARE probes, using cold competition. 50 ng of purified recombinant ZFP36L1 protein was incubated with ^32^P-*BCL2* ARE (lane 2) or ZFP36L1 and 5× (lane 3) and 10× molar (lane 4) excess of unlabeled cold competitor *BCL2* ARE probe. Lane 1: *BCL2* ARE probe only. (C) REMSA analysis of recombinant ZFP36L1 binding to *BCL2* ARE probe, using decreasing amounts of recombinant ZFP36L1 protein. Decreasing amounts of the purified ZFP36L1 protein (50–2.5 ng) were incubated with radiolabeled *BCL2* ARE probe. Lane 1: *BCL2* ARE probe alone; Lane 2, 3, 4, and 5: *BCL2* probe and 50, 25, 5, and 2.5 ng of recombinant ZFP36L1 protein respectively. The REMSA experiments described above were repeated at least twice with similar results. (D) Western blot analysis of ZFP36L1/L2 protein expression in non-induced and PMA-induced Ramos cells. Ramos B cells were serum-starved for 16 hours either unstimulated or stimulated with 50 nM PMA for 3 hours Lane 1: Cell lysate from non-induced Ramos cells; Lane 2: Cell lysate from PMA-induced Ramos cells. Anti-human ZFP36L1/L2 antibody (Cell Signaling Technology rabbit-anti- human ZFP36L1/L2 antibody) was used to detect ZFP36L1/L2 expression. An anti-HSP90 antibody was used as a control for protein loading on the gel. The data shown is representative of similar results obtained in two independent experiments. (E) PMA-induced lysates (30 µg) of Ramos B cells were incubated with radiolabeled *BCL2* ARE and 80 ng of anti-ZFP36L1 antibody (Cell Signaling Technology rabbit-anti- human ZFP36L1/L2 antibody) and non-specific (rabbit IgG) antibodies respectively. Lane 1: *BCL2* ARE probe alone; Lane 2: *BCL2* ARE probe incubated with PMA-induced Ramos lysate; Lane 3: *BCL2* ARE probe incubated with PMA-induced Ramos lysate and anti-ZFP36L1/L2 antibody; Lane 4: *BCL2* ARE probe incubated with PMA-induced Ramos lysate and non-specific control antibody. * indicates specific bands.

We focussed on experimental validation of the well-characterised BCL2 protein as a target (shared by both ZFP36 and ZFP36L1 - see Fig. 2B) with ZFP36L1. To this end, we first produced recombinant human His-tagged ZFP36L1 protein in *E. coli* and purified it from inclusion bodies by nickel column chromatography (Fig. S1). Recombinant expressed ZFP36L1 protein was used in RNA electrophoretic mobility shift assay (REMSA) experiments to test whether ZFP36L1 interacted with the *BCL2* ARE. Fig. 3 shows the results of these experiments. A previously characterised *BCL2* ARE probe (see Methods) effectively formed a complex with ZFP36L1 protein (Fig. 3A). Inclusion of a specific antibody to ZFP36L1 inhibited complex formation. Non-specific (control) antibody did not affect levels of complex formation with *BCL2* ARE. Fig. 3B shows the effect of increasing amounts of cold competitor *BCL2* ARE (lanes 2–4) on ZFP36L1/*BCL2* ARE complex formation. Increasing concentrations of cold competitor *BCL2* ARE probes decreases levels of complex formation in a dose-dependent manner. Fig. 3C shows the dose response relationship between recombinant ZFP36L1 protein input and complex formation. Complex formation was lost when 5 ng or less of recombinant ZFP36L1 protein was included in the REMSA reaction.

In further experiments, we investigated whether endogenous ZFP36L1 could also be shown to bind to the *BCL2* ARE. For these experiments, cell lysates from Ramos Burkitt lymphoma B cells, which express high levels of ZFP36L1 in response to PMA stimulation, were used. Fig. 3D shows levels of ZFP36L1 expression in Ramos cells before and 3 hours after stimulation with PMA (30 nM). ZFP36L1 expression is seen as a series of bands, representing different structural forms of the protein at 40–45 kD range on the Western blot. Levels of ZFP36L1 protein were low before stimulation with PMA but were increased several-fold following PMA stimulation. Fig. 3E shows results from a REMSA assay carried out using cell lysates from PMA stimulated Ramos cells. Complex formation was seen using protein lysates from PMA stimulated cells. Addition of anti-ZFP36L1 antibody to the REMSA reactions reduced levels of complex formation whereas inclusion of a non-specific control antibody did not affect levels of complex formation (Fig. 3E). REMSA analyses therefore shows that ZFP36L1 interacts with the *BCL2* ARE.

**Figure 4:**
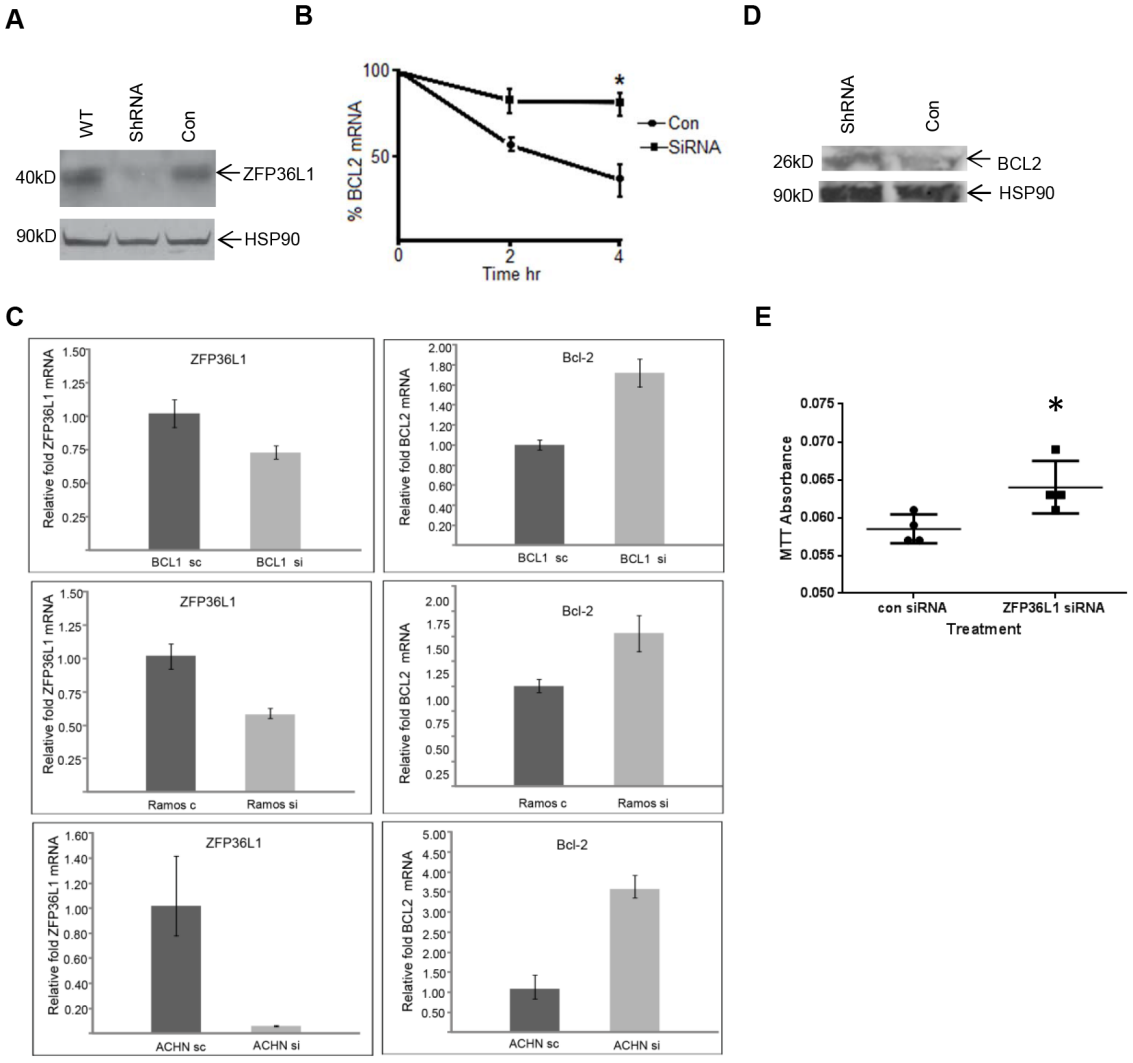
Modulation of ZFP36L1 expression levels is associated with changes in *BCL2* mRNA levels in different cell types. (A) Western blot analysis showing Zfp36L1 protein expression in wild-type, ZFP36L1 ShRNA and control empty lentivirus transduced BCL1 cells. Cell Signaling Technology rabbit-anti-human ZFP36L1/L2 antibody was used to detect Zfp36l1 expression. An anti-HSP90 antibody was used as a control for protein loading on the gel. (B) *BCL2* mRNA degradation analysis following actinomycin D treatment in Zfp36l1-knockdown and control murine leukemia BCL1 cells. qRT-PCR analysis of *Bcl2* mRNA levels over a 4 hour time course in control (empty lentivirus transduced BCL1 cells) and zfp36l1-knockdown BCL1 cells. Cells were treated with 5 µg/ml of actinomycin D for 0, 2, and 4 hours. The results are from 3 independent experiments and show mean ±SEM. * = p<0.05 as determined by student T test. (C) *ZFP36L1* and *BCL2* mRNA levels were measured by qRT-PCR in BCL1, ACHN and Ramos cells. Lentiviral mediated ZFP36L1 ShRNA was used to knockdown *ZFP36L1* mRNA levels in BCL1, ACHN and Ramos cells. qRT-PCR analysis was carried out as described in Methods. Control cells were transduced with lentivirus containing scramble sequence (BCL1, ACHN) or empty lentivirus (Ramos). Mean ±SEM for triplicate samples are shown. These results are representative of at least 2 independent experiments done on each cell type. (D) BCL2 protein expression in ZFP36L1 ShRNA transduced or empty lentivirus (con) transduced Ramos cells. An anti-HSP90 antibody was used as a control for protein loading on the gel. (E) Following either ZFP36L1 or control siRNA oligonucleotide transfection, Ramos cells were treated with ionophore (A23187, 150 nM) and, 24 h later, MTT levels were measured. The results shown are from 4 independent experiments, mean ±SEM are shown. * = p<0.05 as determined by student T test.

Evidence for a role for ZFP36L1 in *BCL2* mRNA degradation came from mRNA degradation analysis following actinomycin D treatment in two different murine leukemia BCL1 cell lines, one of which had been stably transduced with a lentiviral vector expressing Zfp36l1-ShRNA and a second BCL1 cell line that had been stably transduced with control (empty vector) lentivirus [9]. Fig. 4A shows effective knockdown of Zfp36l1 protein expression in the ShRNA transduced BCL1 line whereas control lentivirus expressing cells had equivalent levels of Zfp36l1 expression to wild-type BCL1 cells. Following actinomycin D treatment, *BCL2* mRNA degradation was monitored by qRT-PCR analysis. The half-life of *BCL2* mRNA was estimated to be approximately 2.6 hours in control cells; degradation was significantly delayed in Zfp36l1 ShRNA BCL1 cells (Fig. 4B).

The effect of *ZFP36L1* mRNA knockdown on *BCL2* expression level was also investigated by lentiviral transduction of ZFP36L1 ShRNA in BCL1 and two other cells lines. Fig. 4C shows levels of *ZFP36L1* mRNA knockdown in BCL1, Ramos human Burkitt lymphoma cells and ACHN human renal carcinoma cells compared to control cells. ACHN cells express high levels of *ZFP36L1* mRNA and appreciable levels are also expressed in BCL1 cells with much lower levels expressed in Ramos cells. Significantly, *BCL2* mRNA levels were correspondingly higher in BCL1, ACHN and Ramos cells in ZFP36L1 ShRNA transduced compared to control cells (Fig. 4D). As a positive control mRNA target for ZFP36L1 knockdown, we also observed higher levels of mRNA for the previously published ZFP36L1 target, IL-3 [1], in ZFP36L1 ShRNA transduced BCL1 cells compared to control BCL1 cells (Fig. S2).

We also confirmed that BCl2 protein expression was higher in ZFP36L1 ShRNA transduced Ramos cells compared to control cells (Fig. 4D). We previously reported that BCL2 over-expression protected Ramos cells from ionophore-induced cell death [32]. SiRNA oligonucleotide mediated ZFP36L1 knockdown also protected Ramos cells from ionophore-induced cell death (Fig. 4E) an effect, we previously observed using an antisense ZFP36L1 construct [10], that is consistent with the increased BCL2 expression seen in ZFP36L1-knockdown cells. We previously reported that ZFP36l1 is highly expressed in human tonsillar germinal centres [9] where BCL2 expression is known to be very low (confirmed in immunohistological analysis – data not shown) consistent with an inverse relationship between expression of these two proteins in a physiological context.

**Figure 5:**
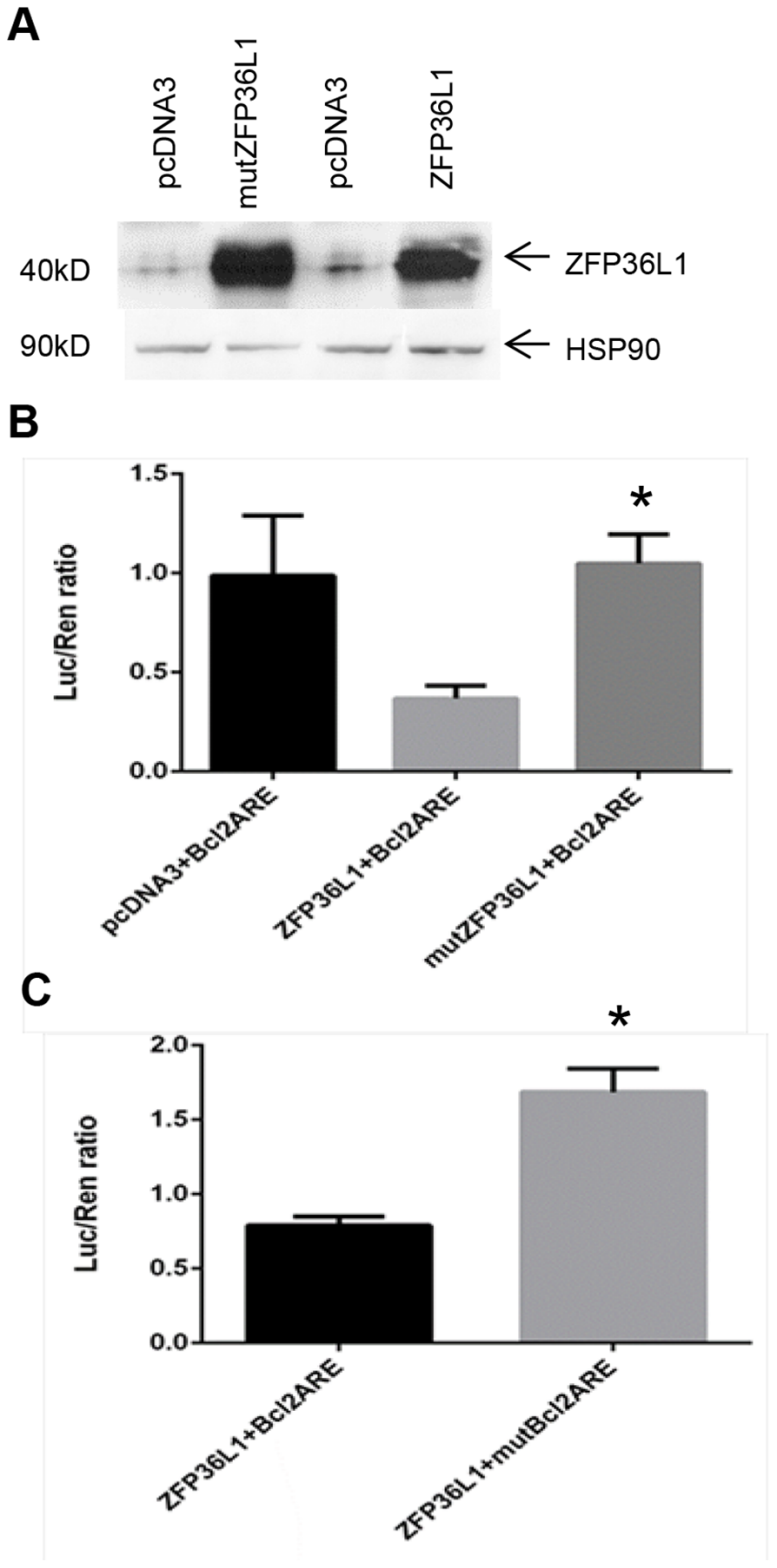
ZFP36L1 mediates degradation of a 3′ UTR *BCL2* ARE. (A) Transfection of ZFP36L1 or mutant ZFP36L1 into HEK293T cells results in high level ZFP36L1 expression as shown by Western blot analysis. Very low/absent levels of ZFP36L1 are found in empty vector (pcDNA3) transfected HEK293T cells. (B) ZFP36L1 interacts with the *BCL2* ARE and mediates *BCL2* ARE degradation. *BCL2* 3′ UTR luciferase reporter assay showing reduced luciferase values in HEK293T cells transfected with ZFP36L1 and *BCL2* ARE compared to values obtained by co-transfection of *BCL2* ARE with either an empty vector (pcDNA3) or a zinc finger mutant ZFP36L1. The results show mean ±SEM for three independent experiments. p<0.05 as determined by student T test. (C) *BCL2* 3′ UTR Luciferase reporter assay showing that ZFP36L1 requires the *BCL2* ARE core AU-rich element to effectively reduce luciferase levels, whereas a *BCl2* ARE construct with the core AU-rich element removed is much less affected. The results show mean ±SEM for three independent experiments, p<0.05 as determined by student T test.

Two *BCL2* ARE constructs (*BCL2* ARE+ and mutant *BCL2* ARE, lacking the AU core binding sequence - see Methods [30]) were cloned into the pmirGlo luciferase reporter vector (Promega). *BCL2* ARE constructs were transfected with either empty vector (pcDNA3), vector containing ZFP36L1 (pcDNA6.ZFP36L1), or vector containing a zinc finger mutant version of ZFP36L1 (pcDNA6.mutZFP36L1) (see Methods). Fig. 5A shows high level expression of both ZFP36L1 and mutZFP36L1 in transfected HEK293T cells compared to vector alone transfected cells. 3′UTR luciferase reporter assays using these constructs showed that in ZFP36L1 transfected cells, luciferase levels were significantly lower than in mutZFP36L1 or empty vector transfected cells (Fig. 5B). Furthermore, removal of the core AU rich region from the *BCL2* ARE significantly inhibited ZFP36L1 mediated degradation of the *BCL2* ARE (Fig. 5C), consistent with this region being important for ZFP36L1 binding to the *BCL2* ARE.

## Discussion

BCL2, a cell survival protein whose dysregulation is a hallmark of a number of malignancies, is controlled by transcriptional, post-transcriptional and post-translational mechanisms (reviewed in [33]). Significantly, three AU-rich-RNA-binding proteins have previously been shown to regulate BCL2 levels via binding to ARE 3′ UTR elements in the *BCL2* mRNA: AUF1 functions to destabilise *BCL2* mRNA [30], [34], nucleolin (acting antagonistically with AUF1) stabilises the *BCL2* mRNA [34]–[36] while HUR acts to both stabilise and enhance translational efficiency of *BCL2* mRNA [37] (reviewed in [2]). Our data reveal a role for a fourth RNA-binding protein, ZFP36L1, in mediating BCL2 post-transcriptional regulation by interacting with and mediating degradation of the *BCL2* mRNA. The zinc finger motifs of ZFP36L1 are evidently required for this interaction. These findings are consistent with previous observations on apoptosis induced by ZFP36 family members, specifically the requirement for a functional zinc finger domain [13], [14] and the apoptosis rescue elicited by over-expression of exogenous BCL2 [14]. Given the important role of BCL2 in normal and malignant B cell survival [33], identification of *BCL2* mRNA as a target for ZFP36L1 may provide a mechanism for a number of experimental observations implicating ZFP36L1 in apoptosis induction in malignant B cells [10], [11]. Although not further investigated in the present study, it is quite likely that *BCL2* mRNA, inferred from regulatory network reconstruction as a target for the ZFP36 protein, is also regulated in an analogous fashion by this ZFP36 family member. Having established the ZFP36L1/BCL2 axis at a mechanistic level, it will be very important to further evaluate the biological importance of this axis in malignant B cell survival and death in future studies.

In addition to AU-rich RNA binding proteins, microRNAs (miRNA), notably miR-15 and miR-16, have also previously been shown to target the *BCL2* mRNA for destruction via binding to AREs [38], [39]. The observation that miR-16 requires the cooperative activity of ZFP36 family members in order to bind to AREs [40] is entirely consistent with our own observations on direct functional binding of ZFP36L1 to the 3′UTR of *BCL2* mRNA. Finally, we note that all four RNA-binding proteins (AUF1, nucleolin, HUR, ZFP36L1) that are now known to target AREs in the *BCL2* 3′ UTR are themselves capable of physical association with each other via protein-protein interaction (see ref [2]). Thus, the coordinate/cooperative activities of multiple RNA-binding proteins that orchestrate the post-transcriptional ‘regulome’ on *BCL2* mRNA probably function in consort with miRNA pathways as components of higher-order macromolecular complexes.

In addition to *BCL2*, the inferred regulatory network for the ZFP36 family proteins constructed in our study identified other mRNAs encoding pro-survival proteins that may also function in the apoptotic response elicited by ZFP36 family members. It is noteworthy that these targets overlapped between different ZFP36 family members. The more global picture of inferred targets (Fig. 2A) also revealed extensive overlap, but with each ZFP36 family member targeting its own unique set of mRNAs. It should be noted that the MIC algorithm that was employed to infer target mRNAs will only identify those that display a strong dependency on the expression of ZFP36 family proteins. While these mRNAs are likely to represent targets that are most strongly regulated by ZFP36 proteins, there will undoubtedly be numerous other mRNAs targets that are subject to dominant regulation of expression via other post-transcriptional and transcriptional mechanisms that are weakly influenced by ZFP36 proteins. Nonetheless, this reconstructed regulatory network is consistent with the partial redundancy of biological functions of the ZFP36 protein family. Whereas all three ZFP36 proteins elicit an apoptosis response (typically observed *in vitro*), subtle differences are evident, particularly in studies using mouse models on other functions related to tumourigenesis. For example, mouse models have provided evidence that ZFP36 family proteins can function as tumour suppressor proteins in lymphoid malignancies. Deletion of *ZFP36L1* and *ZFP36L2* was reported to promote T cell leukemia in knockout mice by targeting *notch1* mRNA [41] Intriguingly, leukemia did not develop in single *ZFP36L1* or *ZFP36L2* knockout mice [41]. In Eμ-Myc mice, a mouse model of human Burkitt lymphoma, and also in human Burkitt lymphoma versus non-Burkitt lymphomas, ZFP36 and ZFP36L1 were shown to be transcriptionally repressed [42]. Moreover, ZFP36, but not ZFP36L1, was shown to function as a tumour suppressor in the Eμ-Myc model [42]. Notwithstanding the lack of tumour suppressor function of ZFP36L1 in the Eμ-Myc mouse model, other studies in human B-lymphoid malignancies have implicated loss of ZFP36L1 function in disease pathogenesis. For example, in a subset of human B cell malignancies there are interstitial deletions of the *ZFP36L1* locus at 14q24 [43] and, recent whole genome sequencing of tumours from multiple myeloma patients has identified regulatory-region and coding-region mutations in the *ZFP36L1* gene [44]. The comprehensive identification of mRNA targets for ZFP36L1 (and for other members of this family) should provide mechanistic insight into the role of these proteins in malignancies of the B-lymphoid and other cell lineages.

## Supporting Information

Figure S1 **Production of bacterially expressed ZFP36L1 protein.** Western blot showing recombinant human ZFP36L1 protein expression Lanes 1, 2 and 3 (*Ve*+): Positive controls containing 5, 1 and 0.2 µl of total protein lysate from BL21 *E.coli* cells, transformed with a recombinant pET15b vector containing the human *ZFP36L1* open reading frame. Lane 4 (*Ve*–): Negative control containing 5 µl of lysates from *E.coli* BL21 cells, transformed with an empty pET15b vector. Lanes 5, 6 and 7: correspond to 5, 10 and 20 µl of ZFP36L1 protein (approximately 100 ng/µl) purified according to the protocol detailed in Materials and Methods. Anti-ZFP36L1 antibody (Cell Signaling Technology rabbit-anti-human ZFP36L1/L2 antibody) was used to detect ZFP36L1 protein.
(TIF)

Figure S2 **IL-3 mRNA levels are increased in BCL1 cells transduced with a ZFP36L1 ShRNA.** IL-3 mRNA levels were measured by qRT-PCR in lentiviral mediated ZFP36L1 ShRNA transduced BCL1 cells (see Fig. 4A) and cells transduced with scramble sequence containing lentivirus. The results show mean ±SEM from 6 replicate samples.
(TIF)

Table S1 **Inferred target mRNAs for ZFP36 family members.**
(XLS)

Table S2 **Enrichment of Gene Ontology terms (Biological Process) in inferred ZFP36 family target mRNAs.**
(XLS)
