# Supplementary figures and images for: Post-Transcriptional Regulation of BCL2 mRNA by the RNA-Binding Protein ZFP36L1 in Malignant B Cells
Source: PLoS One. 2014 Jul 11;9(7):e102625. doi: 10.1371/journal.pone.0102625 (PMC4094554; doi:10.1371/journal.pone.0102625)

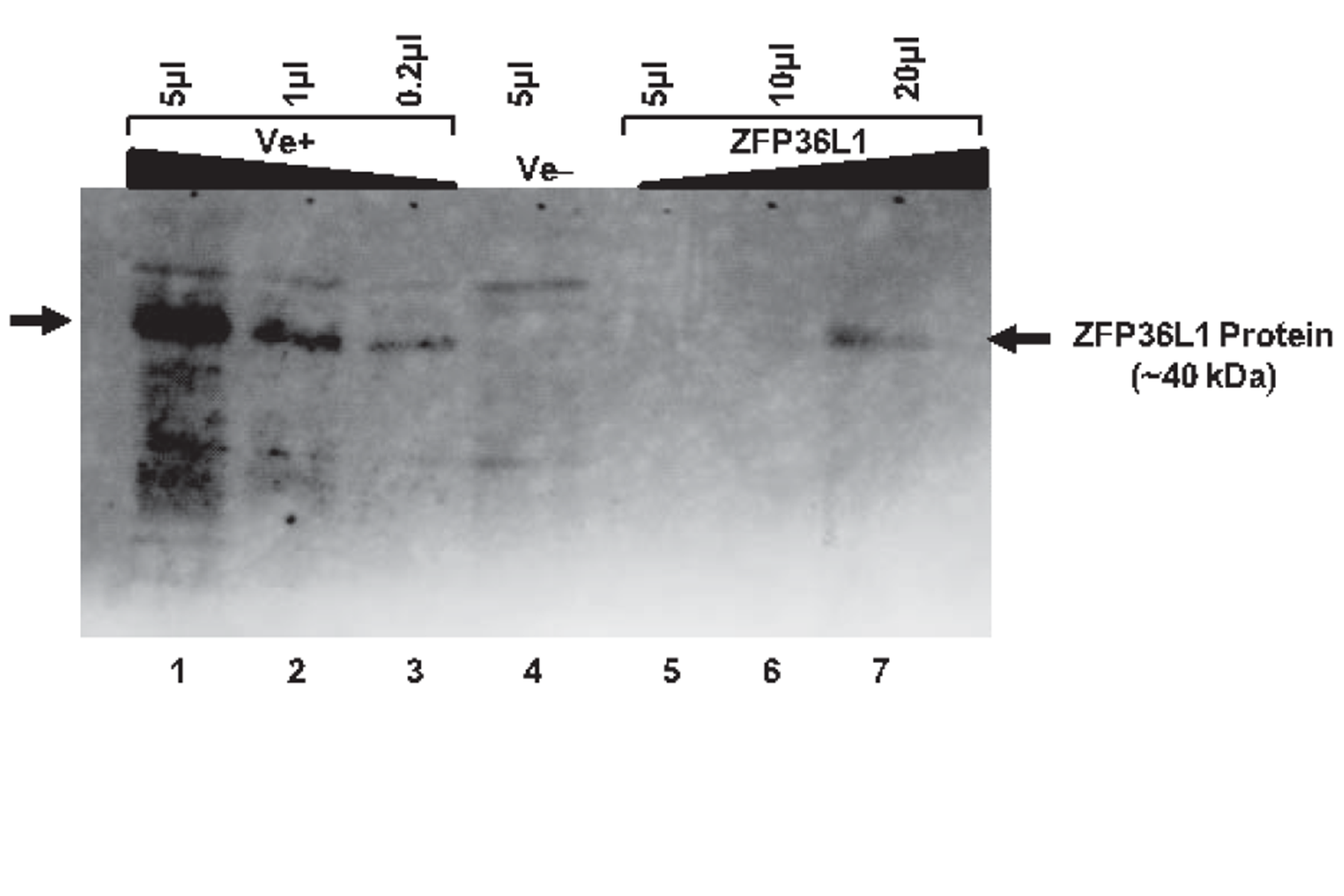

Supplement: Figure S1 — Production of bacterially expressed ZFP36L1 protein. Western blot showing recombinant human ZFP36L1 protein expression Lanes 1, 2 and 3 (Ve+): Positive controls containing 5, 1 and 0.2 µl of total protein lysate from BL21 E.coli cells, transformed with a recombinant pET15b vector containing the human ZFP36L1 open reading frame. Lane 4 (Ve–): Negative control containing 5 µl of lysates from E.coli BL21 cells, transformed with an empty pET15b vector. Lanes 5, 6 and 7: correspond to 5, 10 and 20 µl of ZFP36L1 protein (approximately 100 ng/µl) purified according to the protocol detailed in Materials and Methods. Anti-ZFP36L1 antibody (Cell Signaling Technology rabbit-anti-human ZFP36L1/L2 antibody) was used to detect ZFP36L1 protein. (TIF) [file pone.0102625.s001.tif]

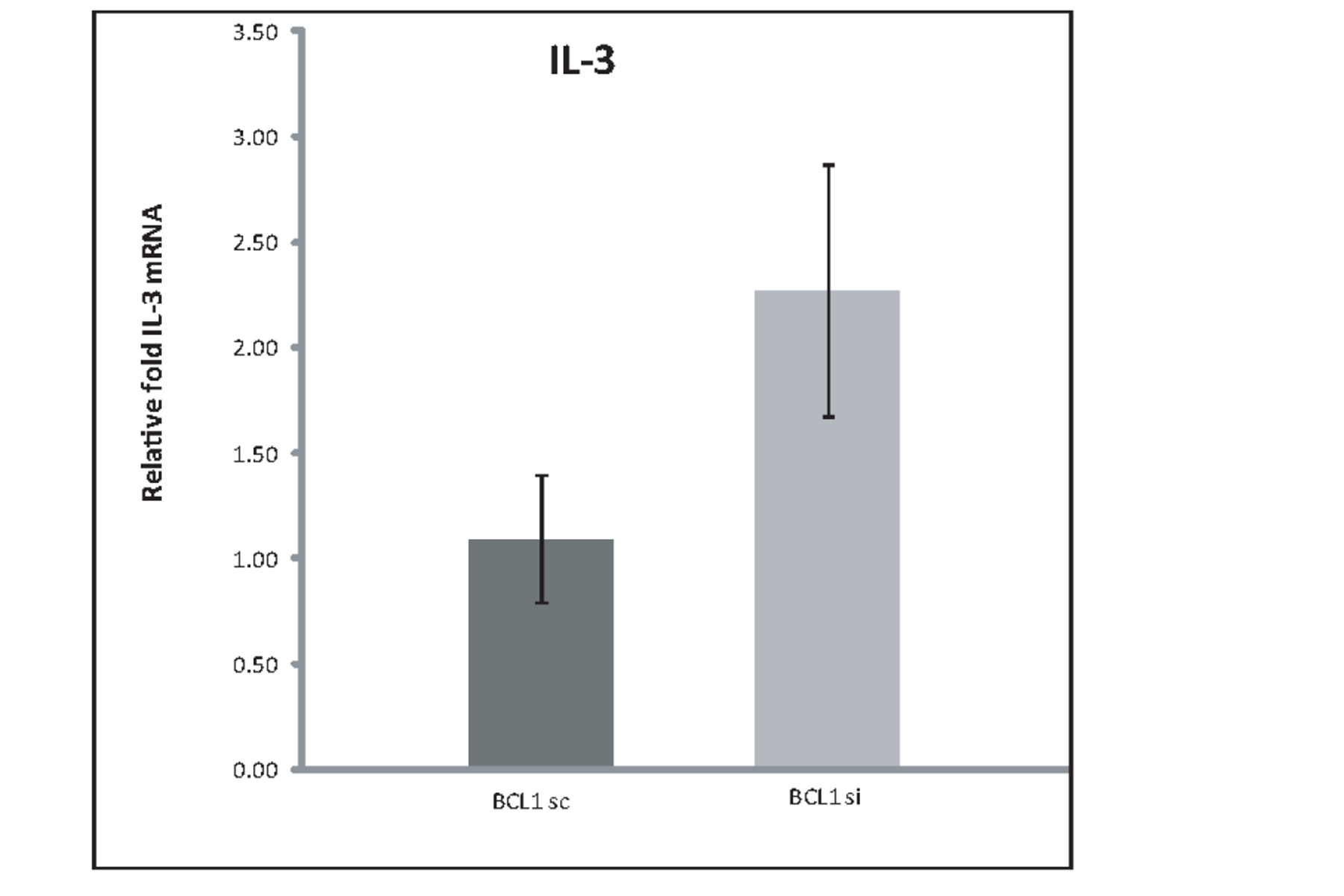

Supplement: Figure S2 — IL-3 mRNA levels are increased in BCL1 cells transduced with a ZFP36L1 ShRNA. IL-3 mRNA levels were measured by qRT-PCR in lentiviral mediated ZFP36L1 ShRNA transduced BCL1 cells (see Fig. 4A) and cells transduced with scramble sequence containing lentivirus. The results show mean ±SEM from 6 replicate samples. (TIF) [file pone.0102625.s002.tif]
